# Consensus Guidelines and Recommendations for the anti-CD38-based Therapy in Clinical Practice for Relapsed/Refractory Multiple Myeloma: From the Pan-Pacific Multiple Myeloma Working Group

**DOI:** 10.46989/001c.141401

**Published:** 2025-08-08

**Authors:** Wenming Chen, Zhen Cai, Chor Sang Chim, Wee Joo Chng, Juan Du, Chengcheng Fu, Wen Gao, Ichiro Hanamura, Jian Hou, Jeffrey Shang-Yi Huang, Tadao Ishida, Chunrui Li, Aijun Liu, Vadim Ptushkin, Naoki Takezako, Raymond Siu Ming Wong, Dok Hyun Yoon

**Affiliations:** 1 Department of Hematology, Myeloma Research Center of Beijing, Beijing Chaoyang Hospital, Capital Medical University, Beijingk, China; 2 Bone Marrow Transplantation Center, the First Affiliated Hospital, Zhejiang University School of Medicine, Zhejiang, China; 3 Department of Medicine, Hong Kong Sanatorium Hospital, Hong Kong; 4 National University Cancer Institute, Singapore; 5 Myeloma & Lymphoma Center, Shanghai Changzheng Hospital, Naval Medical University, Shanghai, China; 6 The First Affiliated Hospital of Soochow University, Jiangsu, China; 7 Department of Hematology, Myeloma Research Center of Beijing, Beijing Chaoyang Hospital, Capital Medical University, Beijing, China; 8 Division of Hematology, Department of Internal Medicine, Aichi Medical University, Aichi, Japan; 9 Department of Hematology, Renji Hospital, School of Medicine, Shanghai Jiao Tong University, Shanghai, China; 10 National Taiwan University Hospital, Taiwan; 11 Japanese Red Cross Medical Center, Tokyo, Japan; 12 Department of Hematology, Tongji Hospital, Tongji Medical College, Huazhong University of Science and Technology, Wuhan, Hubei, China; 13 Botkin Moscow City Clinical Hospital, Russia; 14 Nerima Hikarigaoka Hospital, Tokyo, Japan; 15 Sir Y.K. Pao Centre for Cancer & Department of Medicine and Therapeutics, Prince of Wales Hospital, The Chinese University of Hong Kong, Hong Kong; 16 University of Ulsan College of Medicine, Asan Medical Center, Seoul, South Korea

**Keywords:** Relapsed or refractory multiple myeloma, Anti-CD38 monoclonal antibody, Triplet therapy, Isatuximab, Daratumumab

## Abstract

Anti-CD38 monoclonal antibodies (mAbs), including daratumumab and isatuximab, have become key components of treatment for relapsed/refractory multiple myeloma (RRMM). This expert consensus provides evidence-based guidance on their optimal use, including regimen selection, special considerations for elderly or frail patients, and the treatment of high-risk subgroups. Key topics addressed include the selection of anti-CD38-based regimens, patient stratification by frailty and comorbidities, strategies for managing hematologic toxicities, and considerations for re-treatment. Anti-CD38 mAb-based regimens have demonstrated clinical efficacy across diverse RRMM populations, including patients with high-risk cytogenetic abnormalities such as 1q21+. While resistance remains a clinical challenge, particularly in previously exposed patients, current evidence supports the feasibility of anti-CD38 mAb rechallenge following a substantial washout period (typically 6 to 12 months), which may allow partial recovery of CD38 expression and immune effector function. The consensus also emphasizes the continued utility of these agents in elderly or frail individuals, where durable responses can be achieved with appropriate monitoring and supportive care. Moreover, anti-CD38 mAbs are recognized as key components within evolving treatment paradigms, supporting their use for combination strategies involving emerging immunotherapies such as CAR-T cells and bispecific antibodies. This consensus provides a framework to guide individualized treatment decisions and highlights the need for continued research to optimize the integration of anti-CD38 mAbs into the modern therapeutic landscape of RRMM.

## Introduction

Multiple myeloma (MM) is a progressive B-cell malignancy, accounting for approximately 1%-1.8% of all cancers and ranking as the second most common hematologic malignancy, comprising 10%-15% of hematologic cancers and contributing to 22% of blood and bone marrow cancer-related mortality in 2022.^1–5^ Over the past decades, the therapeutic landscape of newly diagnosed multiple myeloma (NDMM) has evolved significantly, primarily due to the incorporation of immunomodulatory drugs (IMiDs) and proteasome inhibitors (PIs) into combination treatment regimens, reducing early mortality and increasing 5-year overall survival (OS).^6–8^ In recent years, triplet combinations of IMiDs, PIs, and dexamethasone (e.g., VRd, KRd, etc.) remain the standard of care for NDMM in most countries worldwide. More recently, the addition of anti-CD38 monoclonal antibody (mAb), including daratumumab and isatuximab, to these triplet regimens has led to deeper responses and improved outcomes in both autologous stem cell transplant (ASCT)-eligible and -ineligible patients while maintaining a manageable safety profile.^9^ Lenalidomide-based maintenance is standard post-induction, and for transplant-ineligible patients, daratumumab plus lenalidomide and dexamethasone (Dara-Rd) is approved for use as frontline treatment in many countries. Despite these advances, MM remains incurable, with most patients developing refractory disease, defined as nonresponsiveness to primary or subsequent therapy or progression within 60 days of the last treatment.^10^ Furthermore, the majority of patients who initially respond will ultimately experience relapse, necessitating the need for effective treatment strategies for relapsed/refractory multiple myeloma (RRMM).^11,12^

In multiple myeloma, relapse classifications are pivotal for prognostication and therapeutic decision-making. Early relapse in MM, commonly defined as disease progression within 12 to 18 months of initiating therapy or post-autologous stem cell transplantation (ASCT), is associated with aggressive disease biology and poor outcomes.^13–15^ The early relapse patients often exhibit high-risk cytogenetic abnormalities (HRCAs), such as del(17p), gain(1q), t(4,14), t(14;16) or t(14;20), and is associated with a reduced progression free survival (PFS) and overall survival (OS).^16–18^ However, while early relapse provides valuable prognostic information, it is not routinely used as the primary framework for treatment decision-making in RRMM. In clinical practice and international guidelines such as the National Comprehensive Cancer Network (NCCN) Guidelines for Multiple Myeloma (Version 2.2026), European Hematology Association and European Myeloma Network (EHA-EMN) 2025 guidelines and the International Myeloma Working Group (IMWG) guidelines for MM updated in 2024, RRMM management is more commonly stratified by the number of prior lines of therapy and the patient’s refractory status to specific drug classes, particularly IMiDs, PIs, and anti-CD38 mAbs.^19,20^ It is also confirmed that patients with double refractory to bortezomib and lenalidomide exhibit significantly worse outcomes.^21^ These distinctions highlight MM’s heterogeneity and the need for tailored strategies based on timing and prior therapies.

In order to address the unmet clinical requirements of RRMM patients, the spectrum of new agents has greatly expanded in the past decade.^22^ The introduction of anti-CD38 mAbs, such as daratumumab and isatuximab, has significantly transformed the treatment paradigm for RRMM, addressing the unmet need for novel therapeutic strategies. Daratumumab, a human IgG1 mAb, exerts its anti-myeloma activity through multiple mechanisms, including complement-dependent cytotoxicity (CDC), antibody-dependent cellular cytotoxicity (ADCC), antibody-dependent cellular phagocytosis (ADCP), and Fc receptor-mediated crosslinking-induced apoptosis, thereby establishing itself as a key agent in RRMM treatment.^23–27^ Conversely, isatuximab, an IgG1 mAb able to mediate anti-myeloma activity through multiple mechanisms of action, including direct induction of apoptosis without crosslinking, ADCC, ADCP, and CDC, offering an alternative therapeutic option for RRMM.^28–30^ The integration of anti-CD38 mAbs into RRMM treatment regimens has represented a major advancement, with their incorporation into different combinations in conjunction with IMiDs, PIs, dexamethasone, demonstrating significant improvements in clinical outcomes.^31^ The randomized controlled trials (RCTs) incorporating anti-CD38 mAbs with IMiDs and/or PIs, including CANDOR, IKEMA, APOLLO, and ICARIA-MM, have demonstrated promising outcomes for RRMM patients in different risk populations and refractory states, highlighting that anti-CD38-based therapies are efficacious and safe treatment options for patients with RRMM.^32–35^

This consensus paper aims to provide a comprehensive evaluation of anti-CD38-based therapies in the treatment of RRMM. Given the growing body of evidence supporting the efficacy of daratumumab and isatuximab, this document synthesizes clinical trial data, real-world evidence, and expert opinions to guide optimal treatment strategies. It addresses key clinical questions, including the role of anti-CD38 mAbs in different treatment combinations, their efficacy in specific patient subgroups, safety considerations, and the feasibility of retreatment strategies. Furthermore, this consensus integrates findings from major RCTs to compare the relative efficacy and safety of these agents against standard therapies. By offering evidence-based recommendations, this paper aims to assist healthcare professionals in making informed treatment decisions for RRMM patients, ultimately improving clinical outcomes and patient care.

## Materials and Methods

A modified Delphi consensus process was employed to develop statements on the use of anti-CD38 monoclonal antibody-based therapy in the treatment of RRMM. The Delphi method is a well-established, consensus-driven approach that integrates evidence review and expert opinion to guide clinical decision-making.^36^ This modified process involved a combination of anonymous online voting and in-person meetings by a panel of experts. A consensus threshold of 70% agreement was predefined.^37^ While this threshold was chosen to reflect meaningful agreement, it does not imply unanimity. Statements meeting this threshold are referred to as having “consensus” throughout the manuscript. The panel aimed to establish evidence-based recommendations informed by pivotal clinical trials and real-world practice, offering clear guidance on optimizing treatment strategies for RRMM patients across different relapse statuses and special populations. Panelists were selected based on their active involvement in clinical trials and contributions to the multiple myeloma field, ensuring a diverse range of perspectives. The selection process was conducted by an independent planning committee, separate from funding bodies, further reinforcing the credibility of the panel’s conclusions. Finally, 17 hematology and oncology experts from the Asia-Pacific region, all with extensive experience in RRMM management, participated in the consensus development. All experts provided informed consent for their involvement in this process.

The consensus development process included a systematic review of the literature, with a structured search focusing on peer-reviewed studies. The Grading of Recommendations Assessment, Development, and Evaluation (GRADE) system was used to assess the quality of evidence and the strength of recommendations.^38^ Evidence quality was categorized as High (A), Moderate (B), Low, or Very Low (C) based on study design and limitations. The strength of recommendations was classified as either Strong (1) or Weak (2), considering the balance of benefits and harms of the intervention. Key clinical trials, particularly pivotal randomized controlled trials and meta-analyses were prioritized due to their significant contributions to understanding the efficacy and safety of anti-CD38-based regimens. Additionally, guidelines from authoritative bodies such as the NCCN and the IMWG were reviewed to ensure alignment with current clinical practice.

A research steering committee reviewed the findings from the systematic literature review and formulated ten statements for the initial online voting round. These statements covered four key domains: (1) Prioritization of anti-CD38-based therapy for RRMM, (2) Optimization of anti-CD38-based therapy strategies for special populations, (3) Management and monitoring of potential adverse events (AEs) associated with anti-CD38-based therapy, and (4) Future perspectives on combining anti-CD38 antibodies with novel agents for RRMM. In March 2025, experts anonymously voted on these statements, indicating their agreement or disagreement and suggesting revisions. An in-person meeting was held in April 2025 to review the voting results and refine the statements. In May 2025, all participating experts conducted a joint review of the consensus document, which underwent multiple rounds of revision before finalization.

## Results

### Chapter 1: Treating RRMM with anti-CD38-based therapy

Should anti-CD38-based therapy be used for patients with first-relapse RRMM?

#### Statement

Anti-CD38-based therapy should be used for patients with first-relapse RRMM (A1).

#### Discussion

For NDMM, standard treatment typically involves combination therapy with a PI, an IMiDs, and dexamethasone, followed by high-dose melphalan and ASCT, and lenalidomide-based maintenance. In recent years, anti-CD38-based quadruplet therapies (e.g., Daratumumab in combination with bortezomib, IMiDs, and dexamethasone) have become the new standard for transplant-eligible patients. For transplant-ineligible patients, anti-CD38-based triplet therapies (e,g, Dara-Rd) are also implemented. Despite their incorporated into frontline regimens, anti-CD38 mAbs are less widespread in real-world practice compared to traditional triplet combinations (PIs, IMiDs, and dexamethasone). Consequently, resistance discussions in NDMM still centered on PI- and IMiD-based therapies. Prolonged exposure in frontline and maintenance settings commonly leads to lenalidomide and/or bortezomib resistance, requiring alternatives such as anti-CD38 mAbs or B cell maturation antigen (BCMA)-directed therapies. For patients previously exposed to anti-CD38 mAbs, relapse to daratumumab or isatuximab gradually develop with the prolonged exposure.

Lenalidomide-refractory patients usually demonstrate significantly worse outcomes. A pooled analysis of 24 clinical trials and 19 real-world studies demonstrated that lenalidomide-refractory patients had markedly shorter survival compared to the intent-to-treat population, with a median PFS of 8.8 months vs. 13.8 months, and overall survival (OS) of 21.7 months vs. 35.9 months, respectively.^39^ Double refractory patients (to both lenalidomide and bortezomib) had even shorter PFS (5.5 months), underscoring the urgent need for more effective alternative regimens in this double-refractory high-risk group.

Anti-CD38 mAbs are well-suited for RRMM due to their diverse mechanisms, including ADCC, ADCP, CDC, direct apoptosis induction, and immune modulation through depleting immunosuppressive CD38^+^ regulatory cells and ectoenzyme inhibition.^40^ The results enhance T-cell activation and immune surveillance, overcoming resistance to prior therapies. Daratumumab and isatuximab, both approved for NDMM and RRMM, are now recommended by NCCN as preferred treatments due to their proven benefits in PFS and MRD negativity. The phase I/II GEN501 and phase II SIRIUS trial demonstrated that daratumumab monotherapy has favorable efficacy results in heavily pretreated, refractory MM patients.^41,42^ A pooled analysis of 148 patients with a median of five prior lines of therapy, including 86.5% double-refractory to a PI and an IMiD from these two trials demonstrated an overall response rate (ORR) of 31.1%, including very good partial responses (VGPR, defined as > 90% reduction in serum myeloma protein (M-protein) with urine M-protein level < 100 mg/24 h) in 13 patients, and 4 complete responses (CR, defined as negative immunofixation on the serum and urine and disappearance of any soft tissue plasmacytomas and < 5% plasma cells in bone marrow).^43^ Median PFS was 4.0 months (95% confidence interval [CI], 2.8-5.6), and OS was 20.1 months (95% CI, 16.6-not estimable [NE]). Responses were rapid, deep, and durable, with clinical benefit even in patients with stable disease (SD, defined as < 50% reduction in serum M-protein level, and no progressive disease) or better. No new safety issues were identified.

Anti-CD38-based therapies have been investigated due to their synergistic effects with other antineoplastic agents, including IMiDs and PIs, as demonstrated in *in vitro* studies, supporting their role in enhancing therapeutic depth.^44,45^ For patients relapsing after one to three prior therapies, anti-CD38-based regimen selection is guided by refractory status to IMiDs and/or PIs. In bortezomib- or lenalidomide-refractory, phase III trials have demonstrated that combinations including daratumumab, carfilzomib, and dexamethasone (Dara-Kd), isatuximab, carfilzomib, and dexamethasone (Isa-Kd), daratumumab, pomalidomide, and dexamethasone (Dara-Pd), and isatuximab, pomalidomide, and dexamethasone (Isa-Pd) provide effective disease control. The important outcomes of these anti-CD38-based phase III trials are summarized in **Table 1**. Additionally, benefits observed across different subgroups regarding prior therapy exposures and refractoriness are demonstrated in **Table 2**.

**Table 1.** Key Outcomes of Anti-CD38-Based Regimens in Phase III RRMM Trials

*Trial*	*Regimen*	*Median PFS (months)*	*HR (PFS) (95% CI)*	*Median OS (months)*	*HR (OS) (95% CI)*	*MRD Negativity*
CANDOR	Dara-Kd	28.4	0.64 (0.49-0.83) p = 0.0018	50.8	0.784 (0.595-1.033) p = 0.042	28%
	Kd	15.2		43.6		9%
IKEMA	Isa-Kd	35.7	0.58 (0.42-0.79) p < 0.001	NR	0.855 (0.608-1.202) p = 0.18	33.5%
	Kd	19.2		50.6		15.4%
APOLLO	Dara-Pd	24.4	0.73 (0.55-0.98) p = 0.034	34.4	0.82 (0.61-1.11) p = 0.20	9%
	Pd	17.6		23.7		2%
ICARIA-MM	Isa-Pd	11.5	0.60 (0.44-0.81) p < 0.001	24.6	0.76 (0.57-1.01) p = 0.028	5.2%
	Pd	6.5		17.7		0%

Dara, daratumumab; Isa, isatuximab; Kd, carfilzomib + dexamethasone; Pd, Pomalidomide + dexamethasone.

**Table 2.** Subgroup Analysis of PFS by Refractory Status in Phase III RRMM Trials

*Trial*	*Regimen*	*Subgroup* *(Prior Treatment / Lines)*	*Median PFS (months)*	*Median PFS (Control) (months)*	*HR (95% CI)*
ICARIA-MM	Isa-Pd VS Pd	Lenalidomide-refractory	11.40	5.59	0.593 (0.431-0.816)
		Last-line lenalidomide-refractory	11.60	5.70	0.50 (0.34-0.76)
		PI-refractory	11.40	5.59	0.578 (0.405-0.824)
		Double refractory to lenalidomide and PI	11.20	4.76	0.579 (0.401-0.835)
		2-3 prior lines	12.26	7.82	0.590 (0.397-0.878)
		> 3 prior lines	9.40	4.27	0.590 (0.356-0.977)
APOLLO	Dara-Pd VS Pd	Lenalidomide-refractory	9.9	6.5	0.66 (0.49-0.90)
		Last-line lenalidomide-refractory	8.3	6.1	0.67 (0.47-0.95)
		PI-refractory	8.3	6.3	0.73 (0.49-1.08)
		Double refractory to PIs and IMiDs	7.7	6.1	0.74 (0.49-1.12)
		2-3 prior lines	10.7	6.5	0.66 (0.48-0.92)
		≥ 4 prior lines	19.3	6.6	0.40 (0.18-0.90)
CANDOR	Dara-Kd VS Kd	Lenalidomide-refractory	28.1	11.1	0.46 (0.28-0.73)
		Bortezomib or ixazomib refractory	13.1	8.7	0.65 (0.42-1.00)
		1 prior line	NE	21.3	0.66 (0.42-1.04)
		2-3 prior lines	24.2	12.5	0.55 (0.39-0.78)
IKEMA	Isa-Kd VS Kd	Lenalidomide-refractory	NE	15.70	0.60 (0.34-1.06)
		Last-line lenalidomide-refractory	NE	16.16	0.69 (0.35-1.39)
		1 prior line	NE	NE	0.59 (0.31-1.12)
		> 1 prior lines	NE	16.16	0.48 (0.29-0.78)

Dara, daratumumab; Isa, isatuximab; Kd, carfilzomib + dexamethasone; NE, not evaluable; Pd, Pomalidomide + dexamethasone.

In phase III CANDOR trial, Dara-Kd significantly improved PFS(28.4 vs. 15.2 months; hazard ratio [HR], 0.64) and showed a favorable trend in OS (50.8 vs. 43.6 months; HR, 0.78; P = 0.042) compared with carfilzomib and dexamethasone (Kd), particularly in high-risk cytogenetics (34.3 vs. 17.1 months; HR, 0.52) and lenalidomide- and PI-refractory patients. Higher VGPR or better (69% vs. 47%) supported deeper responses.^32,46^

Similarly, the IKEMA trial showed that Isa-Kd prolonged PFS (35.7 vs. 19.2 months; HR, 0.58), improved MRD negativity (33.5% vs. 15.4%), and greater CR rates (44.1% vs. 28.5%) than Kd, with consistent benefit in lenalidomide-refractory and >1 prior-line subgroups.^33,47^ The regimen delayed time to next treatment (HR, 0.55) and improved PFS2 (defined as the time from randomization to disease progression on next-line treatment or death, whichever occurred first) (HR, 0.68), demonstrating prolonged disease control. Although the OS benefit did not reach statistical significance, there was a favorable trend, with 48-month OS rates of 59.7% vs. 52.2% in the control arm.^33^

The APOLLO trial demonstrated that subcutaneous (SC) daratumumab plus pomalidomide and dexamethasone (Pd) significantly improved PFS compared to Pd alone (24.4 vs. 17.6 months; HR, 0.73), with a favorable trend in OS (34.4 vs. 23.7 months; HR 0.82).^34,48,49^ Notably, most patients were lenalidomide-refractory (79.6%), and 42.4% were double-refractory.^48^ Dara-Pd also prolonged PFS2 (median, 24.4 vs. 17.6 months; HR, 0.73; P = 0.0340) and VGPR or better (51% vs. 30%), reflecting deeper responses.^34,49^ Despite with higher neutropenia rates (69% vs. 51%), the safety profile was manageable. These findings reinforce Dara-Pd as an effective regimen for RRMM, especially in lenalidomide- and PI-refractory patients.

The ICARIA-MM trial demonstrated that Isa-Pd significantly improved PFS (11.5 vs. 6.5 months; HR 0.60) and OS (24.6 vs. 17.7 months; HR 0.76) versus Pd in RRMM.^35,50,51^ Isa-Pd also significantly improved the ORR (60% vs. 35%) and rates of deep response, with VGPR or better achieved in 32% vs. 9% of patients, indicating a higher likelihood of durable disease control. Despite higher rates of grade 3-4 neutropenia (50% vs. 35%), the safety profile remained manageable. Subgroup analyses confirmed consistent benefit across lenalidomide-, PI-, and double-refractory populations, as well as those with >3 prior lines of therapy.^52–54^

These findings, supported by NCCN Guidelines, confirm anti-CD38-based therapies as preferred regimens for RRMM, particularly in patients with prior treatment resistance.

#### Conclusion

Anti-CD38-based therapy should be used for patients with first-relapse RRMM without refractory to anti-CD38 mAbs.

#### Level of Consensus

100% (17) agree.

Total: 17 voters.

Are anti-CD38-based quadruplet regimens recommended for patients with RRMM?

#### Statement

Anti-CD38-based quadruplet regimens are suggested for patients with RRMM (B2).

#### Discussion

In the phase II LYRA study (NCT02951819), 14 RRMM patients not refractory to any PI or IMiDs received daratumumab in combination with cyclophosphamide, bortezomib, and dexamethasone (Dara-CVd).^53,54^ Among them, VGPR or better was achieved in 71.4% (10 patients) by the end of induction, with a CR rate improved to 64.3% by the end of the study. The median PFS was 21.7 months (95% CI, 6.8-NE), with an estimated 36-month PFS rate of 31.7% (95% CI, 5.6-63.4). Median OS was NR, with an estimated 36-month OS rate of 50.0% (95% CI, 22.9-72.2). Grade 3-4 treatment-emergent AEs (TEAEs) occurred in 57.1% of patients, mostly neutropenia (21.4%) and leukopenia (14.2%). Infusion-related reactions (IRRs) occurred in 57.1% of patients, but all were grade 1-2. These findings indicate that Dara-CVd followed by daratumumab maintenance showed favorable efficacy and tolerability in RRMM.

A phase I/II study (NCT01665794) of daratumumab in combination with carfilzomib, pomalidomide, and dexamethasone (Dara-KPd) in RRMM demonstrated deep and durable responses with manageable toxicity, even in a high-risk, lenalidomide-refractory population.^52^ Among patients in the Dara-KPd cohort (82% lenalidomide-refractory; 25% PI-refractory; 64% with high-risk cytogenetics), the ORR was 89%, and PFS and OS were not reached at a 26-month median follow-up. The rate of MRD negativity (sensitivity threshold 10⁻⁵) was 65%, with 42% achieving sustained MRD negativity for at least one year. Compared to KPd alone (ORR 86%, median PFS 13 months), Dara-KPd yielded higher CR rates (57% vs. 20%) and deeper responses. In this phase I/II study, dose-limiting toxicities were mainly asymptomatic cytopenias without treatment-related deaths, and the maximum tolerated dose was established as carfilzomib 20/27 mg/m² plus pomalidomide 4 mg. Hematologic adverse events, particularly neutropenia, were more frequent with Dara-KPd compared to KPd, though serious infections remained comparable. Non-hematologic toxicities such as fatigue, diarrhea, and low-grade cardiovascular events were common but manageable. Dara-KPd demonstrated numerically higher response depth and MRD negativity than Dara-Kd (CR ≥57% vs. NR; MRD negativity 65% vs. 28%) and Dara-Pd (MRD negativity 65% vs. 9%). However, cross-trial comparisons are limited by differences in baseline characteristics, including higher proportions of high-risk and lenalidomide-refractory patients in the Dara-KPd study. Nonetheless, Dara-KPd appears promising for heavily pretreated, high-risk RRMM patients.

Ongoing clinical trials are evaluating isatuximab-based quadruplet regimens, including isatuximab with carfilzomib, pomalidomide, and dexamethasone (Isa-KPd, NCT04287855), isatuximab with belantamab mafodotin, carfilzomib, and dexamethasone (NCT05922501), and isatuximab with elotuzumab, pomalidomide, and dexamethasone (Isa-EPd).^55,56^ The NCCN Guidelines for MM recommend the triplet regimens of KPd and elotuzumab, pomalidomide, and dexamethasone (EloPd) as effective options for RRMM, reinforcing their clinical efficacy and established role in RRMM management. Additionally, isatuximab has already been approved in combination with pomalidomide and dexamethasone (Isa-Pd) based on the ICARIA-MM trial, providing a strong rationale for expanding its use into quadruplet regimens with KPd or EPd to further enhance treatment efficacy. Moreover, the clinical success of daratumumab-based quadruplets further supports the rationale for developing isatuximab-based quadruplets to enhance efficacy in RRMM.

#### Conclusion

Anti-CD38-based quadruplet regimens are suggested for patients with RRMM.

#### Level of Consensus

76% (13) agree; 18% (3) neutral; 6% (1) disagree.

Total: 17 voters.

Are different anti-CD38-based regimens suitable for rechallenging patients who received CD38-based treatment only during induction therapy, without maintenance?

Is a washout period of approximately 6 to 12 months required before considering rechallenge with a different anti-CD38 mAb in patients previously treated during induction?

#### Statement

Different anti-CD38-based regimens are suitable for rechallenging patients who received CD38-based treatment only during induction therapy and without maintenance (B2). In addition, the reintroduction of different anti-CD38 mAbs preferable only after a substantial washout period of approximately 6 to 12 months from the last anti-CD38 mAb dose (B2).

#### Discussion

The introduction of anti-CD38 mAbs combined with IMiDs and/or PIs in NDMM has significantly improved depth of responses, higher MRD negativity rates, and prolonged PFS and OS across both transplant-eligible and transplant-ineligible populations.^57^ However, with inevitable relapse and increased number of patients refractory to IMiDs, PIs, and anti-CD38 mAbs, effective strategies following anti-CD38 mAb exposure remain an urgent unmet need.^58^ Rechallenge with a different anti-CD38 mAb, such as isatuximab after daratumumab, is under investigation, although data are still limited and sample sizes small.

One resistance mechanism involves the downregulation of CD38 post-treatment, with re-expression observed around 6-12 months after discontinuation.^59^ Clinical studies suggest that patients who had a longer washout period (≥6 months) exhibited better response rates compared to those rechallenged less than 3 months.^60–62^ The first trial investigating Isa-Pd rechallenge was conducted on 9 heavily treated daratumumab-refractory patients. All subjects previously received lenalidomide and 1 subject received pomalidomide. The trial demonstrated a 77% response rate of MR (defined as 25-49% reduction in the level of the serum M-protein maintained for a minimum of 6 weeks) or better.^63^ In a phase II trial (NCT02514668) including 32 daratumumab-refractory patients, isatuximab monotherapy yielded a disease control rate (DCR, defined as ≥ MR or SD lasting ≥ 8 weeks) of 37.5%.^61^ The majority (62.5%, n = 20) had less than six months between their last daratumumab dose and the first isatuximab administration. Notably, patients with a ≥ 6-month washout period from daratumumab demonstrated a higher DCR (58.3%) compared to 28.6% in those with < 3 months washout. Patients with CD38 receptor density (RD) ≥ 150,000/cell had a higher DCR (66.7%) compared to 37.5% in those with CD38 RD < 150,000/cell. Despite the small sample size, longer washout intervals (≥ 6 months) may improve outcomes, and CD38 RD could help predict response. A multicenter real-world study of 51 daratumumab-refractory RRMM patients treated with Isa-Pd demonstrated an ORR of 56.9%, including 13.7% achieving CR or better and 27.5% achieving VGPR or better. Median PFS was 5.8 months, and median OS reached 21 months. Notably, efficacy was observed regardless of prior daratumumab exposure duration or timing, suggesting that Isa-Pd remains a viable option following daratumumab refractory.^64^

A retrospective study assessing the clinical outcomes of 39 RRMM patients who received isatuximab following prior daratumumab therapy was conducted in Japan.^62^ Of the 39 enrolled subjects, 28 were daratumumab-refractory, and 24 were triple-class (daratumumab/PIs/IMiDs) refractory. As expected, the ORR was significantly higher in patients without than with daratumumab-refractory disease (91% vs. 40%, P < 0.001). The median PFS in patients with and without daratumumab-refractory disease was 5.1 months (95% CI, 3.7-8.0) and NR (95% CI, 4.1 months-NR), respectively (P = 0.007). The median PFS in patients with and without triple-class refractory disease was 5.1 (95% CI, 3.3-6.4) months and NR (95% CI, 4.8 months-NR), respectively (P = 0.001). Among patients with daratumumab-refractory disease, an interval of ≥ 3 months between the last dose of daratumumab and the first dose of isatuximab was associated with significantly prolonged PFS (P = 0.041). The results suggested that a longer washout period may restore treatment effects of anti-CD38 mAbs.

Studies analyzing MM cells from RRMM patients suggest that prolonged anti-CD38 mAb exposure during post-ASCT maintenance may reduce CD38 expression on myeloma cells, limiting the efficacy of subsequent anti-CD38 therapies.^60^ In contrast, patients with a longer drug-free interval after induction may show CD38 re-expression and improved sensitivity to rechallenge. These findings underscore the importance of treatment sequencing and suggest that anti-CD38 retreatment may be more effective after a sufficient washout period, potentially serving as a bridge to definitive therapies such as chimeric antigen receptor (CAR)-T cells or bispecific antibodies (BsAbs).

#### Conclusion

Different anti-CD38-based regimens are suitable for rechallenging patients who received CD38-based treatment only during induction therapy and without maintenance.

#### Level of Consensus

70% (12) agree; 24% (4) neutral; 6% (1) disagree.

Total: 17 voters.

#### Conclusion

In addition, the reintroduction of different anti-CD38 mAbs preferable only after a significant washout period of approximately 6-12 months since the last anti-CD38 mAb dose.

#### Level of Consensus

76% (13) agree; 24% (4) neutral.

Total: 17 voters.

### Chapter 2: Optimization treatment strategy in special populations

#### Statement

Anti-CD38-based therapies are preferred for RRMM patients with impaired renal function (e.g., eGFR between 30 and 60 mL/min/1.73 m²) (A1).

#### Discussion

Are anti-CD38-based therapies preferred for RRMM patients with impaired renal function (e.g., eGFR between 30 and 60 mL/min/1.73 m²)?

Renal impairment is a frequent complication in MM, affecting approximately 20%-61% of patients at diagnosis and approximately 2%-4% of MM patients requiring renal dialysis treatment with disease progression.^65–69^ The malignant plasma cells secrete an excess of immunoglobulins and free light chains of immunoglobulin (Ig)G, which together accumulate in the bloodstream and urine of patients with MM, resulting in renal failure.^70^ It is associated with poorer survival outcomes and higher mortality risks.^71^ In patients with RRMM, renal function further declines due to cumulative treatment exposure, progressive disease, and persistent monoclonal light chain burden. Therefore, the need for dose adjustments and nephrotoxicity concerns limits treatment options, necessitating effective and renally safe therapies.

Anti-CD38 mAbs, including daratumumab and isatuximab, have demonstrated renal safety and efficacy when combined with pomalidomide (Pd) or carfilzomib (Kd), offering superior PFS and ORR compared to standard regimens.^33,35,46,47,49^ The NCCN Guidelines now recommend these regimens as preferred options in patients with an estimated glomerular filtration rate (eGFR) of 30-60 mL/min/1.73 m².

Clinical trials and meta-analyses support the efficacy of anti-CD38-based therapies in RRMM patients with renal impairments. In ICARIA-MM, Isa-Pd significantly improved PFS (11.5 vs. 6.5 months; HR, 0.596) and ORR (60.4% vs. 35.3%) than Pd, even in patients with renal impairment (55 [35.7%] in Isa-Pd vs. 49 [32.0%] in Pd).^35^ Similarly, the APOLLO trial demonstrated superior PFS with Dara-Pd (12.4 vs. 6.9 months; HR, 0.63) than Pd in the renal-impaired subgroup.^34,48,49^

A meta-analysis of 10 randomized controlled trials found that daratumumab-based regimens improved PFS (HR, 0.46; 95% CI: 0.37-0.58) and OS (HR, 0.68; 95% CI: 0.51-0.92) in patients with renal insufficiency.^72^ Additionally, another meta-analysis of anti-CD38-based therapies confirmed consistent PFS (pooled HR, 0.46; 95% CI, 0.37-0.57; P < 0.001) and OS (pooled HR, 0.70; 95% CI, 0.57-0.88; P = 0.002), with no evidence of heterogeneity, further supporting the robust efficacy of both anti-CD38 mAbs in renal-impaired population.^73^ Furthermore, a real-world cohort study evaluating pomalidomide-based regimens in RRMM patients with chronic kidney disease confirmed comparable renal response rates (approximately 50%) in both moderate and severe chronic kidney disease groups, reinforcing the renal safety of pomalidomide when combined with anti-CD38 therapy.^74^ This reinforces anti-CD38 therapy’s effectiveness regardless of renal function status.

While carfilzomib-related nephrotoxicity remains a concern,^75^ evidence suggests anti-CD38 mAbs plus carfilzomib regimens are effective and feasible in RRMM patients with renal impairment. In IKEMA, Isa-Kd prolonged PFS to 35.7 months vs. 19.2 months with Kd (HR, 0.58), with similar benefits in patients with renal impairment (43 [26.1%] and 18 [16.2%] subjects in Isa-Kd and Kd, respectively).^47^ A subgroup analysis demonstrated a significant PFS benefit (HR, 0.27; median PFS was NR vs. 13.4 months with Kd alone), higher renal response rate of 52.0% vs. 30.8%, and durable renal responses lasting ≥60 days occurred in 32.0% vs. 7.7% of patients in the Isa-Kd and Kd groups in patients with impaired renal function (eGFR <60 mL/min/1.73 m²).^76^ In the renal impaired subgroup, the ORR (93.1% vs. 61.1%) and MRD negativity (30.2% vs. 11.1%) were also higher in Isa-Kd compared with Kd, with comparable grade ≥3 AE rates (79.1% vs. 77.8%).

The CANDOR trial also demonstrated improved median PFS with Dara-Kd (28.4 vs. 15.2 months; HR, 0.64), including patients with renal impairment (38 [12.2%] and 27 [17.5%] in Dara-Kd and Kd, respectively).^32^ Rates of acute renal failure were comparable between arms (8.1% vs. 9.2%). Additionally, the ENDEAVOR trial demonstrated that carfilzomib did not worsen renal function compared to bortezomib in renal impaired patients.^77^ Collectively, these findings suggest that while carfilzomib-associated nephrotoxicity remains a consideration, its use in patients with renal impairment is feasible with appropriate monitoring and supportive care.

Given the clinical data supporting anti-CD38-based regimens, the consensus recommends Isa-Pd or Dara-Pd as preferred choices for RRMM patients with renal impairment, due to their renal safety profile and efficacy. For patients requiring a PI-based regimen, Isa-Kd or Dara-Kd are viable options for patients requiring a PI-based regimen, with appropriate monitoring.

#### Conclusion

Anti-CD38-based therapies are preferred for RRMM patients with impaired renal function (e.g., eGFR between 30 and 60 mL/min/1.73 m²).

#### Level of Consensus

100% (17) agree.

Total: 17 voters.

Are anti-CD38-based therapies appropriate for RRMM patients who are frail or over 75 years old?

#### Statement

Anti-CD38-based therapies are appropriate for RRMM patients who are frail or over 75 years old (A1).

#### Discussion

MM is a hematologic malignancy that predominantly affects elderly individuals, with the highest incidence observed in those aged 65 to 74 years and a median age at diagnosis of 70 years.^78^ Elderly patients represent a heterogeneous population, often presenting with multiple comorbidities, diminished functional capacity, and an increased risk of frailty. These factors can significantly impact treatment decisions and tolerability. Additionally, due to restrictive eligibility criteria in most clinical trials, a substantial proportion of very elderly patients (≥75 years) are excluded from pivotal studies. Even when older patients are included, they tend to be unusually fit and not fully representative of the broader elderly MM population, limiting the generalizability of clinical trial findings.^79^ Based on the conducted phase III studies, daratumumab-based regimens have demonstrated favorable efficacy and controllable AEs in elderly patients with RRMM.

In a subgroup analysis from the CASTOR and POLLUX trials, patients aged ≥75 years experienced significantly prolonged PFS with Dara-Rd (28.9 vs. 11.4 months; HR, 0.27; P = 0.0042) and daratumumab plus bortezomib and dexamethasone (Dara-Vd) (17.9 vs. 8.1 months; HR, 0.26; P = 0.0022) compared with their respective control arms.^80^ The ORR was also higher in the daratumumab-containing arms, demonstrating that elderly patients derive significant clinical benefits. However, hematologic toxicities (anemia, neutropenia, and thrombocytopenia) were slightly increased in elderly patients, with grade 3-4 neutropenia occurring in about 50% of patients receiving daratumumab-based therapy. These toxicities were manageable with dose adjustments, growth factor support, and transfusions when necessary. Similarly, the APOLLO trial, which assessed Dara-Pd vs. Pd in previously treated RRMM, further supports its use in elderly patients. The median PFS in patients aged ≥ 65 years was significantly prolonged (14.2 vs. 7.0 months, HR, 0.55; 95% CI, 0.38-0.81). From a safety perspective, daratumumab-based regimens were generally well tolerated in elderly RRMM patients, with manageable AEs.

Isatuximab-based regimens have demonstrated notable efficacy in elderly RRMM patients, as supported by subgroup analyses of the IKEMA and ICARIA-MM trials. In IKEMA, patients ≥ 70 years treated with Isa-Kd experienced a substantial PFS benefit (HR, 0.36; 95% CI, 0.18-0.75) and deeper responses, including higher rates of ≥ VGPR (73.1% vs. 55.9%), MRD negativity rates (23.1% vs. 11.8%), and CR rates (38.5% vs. 23.5%) compared to Kd.^81^ Similarly, in ICARIA-MM, patients aged ≥75 years had a median PFS of 11.4 vs. 4.5 months (HR, 0.48; 95% CI, 0.24-0.95) with Isa-Pd versus Pd, with Isa-Pd achieving higher ORRs in patients ≥75 years (53.1% vs. 31.0%) and 65-74 years (64.7% vs. 38.9%), confirming a significant survival advantage in these high-risk groups.^82^

The safety profiles of isatuximab-based regimens in elderly patients were manageable. In IKEMA, grade ≥3 TEAEs were more frequent with Isa-Kd, but the rates of serious TEAEs were similar (72.5% vs. 70.6%), and fewer elderly patients discontinued treatment (11.8% vs. 23.5%), suggesting that isatuximab did not significantly increase treatment discontinuation in older patients.^81^ Infection-related complications, including pneumonia and upper respiratory tract infections, were more common in elderly patients but were manageable. In ICARIA-MM, hematologic toxicities such as neutropenia were more frequent in elderly patients receiving Isa-Pd, but reversible with supportive care.^82^ Importantly, health-related quality of life was maintained, supporting the regimen’s suitability in older, potentially frail patients.

Eligibility criteria varied across pivotal anti-CD38-based trials in RRMM, with some studies excluding patients with chronic obstructive pulmonary disease (COPD) or cardiovascular impairments. For example, daratumumab trials (EQUULEUS, APOLLO, and CANDOR) excluded patients with COPD and forced expiratory volume in 1 second (FEV1) <50% or moderate to severe persistent asthma,^46,49,83–85^ whereas isatuximab trials (ICARIA-MM and IKEMA) included those with respiratory comorbidities.^82,85^ These differences may influence the generalizability of results. In clinical practice, it is important to recognize that patients with severe respiratory or cardiac conditions should only receive anti-CD38-based therapies with appropriate monitoring supportive measures, such as bronchodilator prophylaxis in patients with COPD receiving daratumumab.

Overall, the evidence from IKEMA and ICARIA-MM supports the use of isatuximab-based regimens in elderly RRMM patients, providing meaningful survival benefits and a manageable safety profile. Isa-Kd and Isa-Pd provide deepened responses and prolong PFS, with clinical benefits in patients with comorbidities and frailty, populations often underrepresented in clinical trials. Compared with daratumumab-based regimens, isatuximab trials allowed broader inclusion criteria, such as patients with respiratory comorbidities, which may enhance their real-world applicability. Similarly, the APOLLO study demonstrated that Dara-Pd was effective and well tolerated in older patients (≥75 years), with a consistent PFS benefit and low discontinuation rates. Although formal frailty scoring was not part of APOLLO, the inclusion of patients with reduced performance status and age-related vulnerabilities, along with consistent benefit across ECOG subgroups, supports the regimen’s suitability for frail populations. The subcutaneous formulation of daratumumab also offers practical advantages, including shorter administration time and fewer infusion-related reactions, making it convenient for elderly or frail patients. Together, these findings support both isatuximab- and daratumumab-based regimens as appropriate and tolerable options for older or frail RRMM patients.

#### Conclusion

Anti-CD38-based therapies are appropriate for RRMM patients who are frail or over 75 years old.

#### Level of Consensus

100% (17) agree.

Total: 17 voters.

Are anti-CD38-based therapies appropriate for RRMM patients with 1q21^+^ abnormalities?

#### Statement

Anti-CD38-based therapies are appropriate for RRMM patients with 1q21^+^ cytogenetic abnormalities (A1).

#### Discussion

The updated IMWG consensus defines high-risk MM based on cytogenetic profiles, such as t(4;14), del(17/17p), t(14;16), t(14;20), non-hyperdiploidy, and 1q gain(1q21^+^), all associated with poor prognosis.^86^ Among these, 1q21^+^, including gain (three copies) and amplification (≥4 copies), is one of the most common chromosomal alterations.^87^ 1q21 gain has been detected in 28%-49% of patients with NDMM and in 42%-80% of those with RRMM, indicating its association with disease progression and treatment resistance.^87–90^ Additionally, its frequent co-occurrence with other high-risk lesions worsens prognosis, posing significant treatment challenges.^91,92^ Prognosis varies with treatment choice, and effective strategies include PIs with lenalidomide/pomalidomide, double ASCT with bortezomib, and anti-CD38 mAbs combined with IMiDs.

For daratumumab-based regimens, evidence regarding efficacy in 1q21^+^ RRMM patients remains limited, as randomized controlled trials such as POLLUX and CASTOR did not specifically report 1q21^+^ subgroup analyses.^93^ A retrospective analysis found that daratumumab-based combinations demonstrated comparable responses in RRMM patients regardless of 1q21^+^.^94^ However, another retrospective study argued that the magnitude of PFS benefit from daratumumab was reduced in those with ≥4 copies of 1q21.^95^ Furthermore, a study including 81 RRMM patients suggested that while daratumumab regimens are effective, patients with concurrent high-risk features (e.g., del(17p) and 1q21 amplification) had inferior outcomes.^96^ These findings suggest that while daratumumab-based therapies remain viable options, it has been speculated that isatuximab-based combinations may offer stronger disease control in patients with 1q21^+^ RRMM, particularly those with amplification (≥4 copies), although further studies are needed to validate this observation.^93^

Isatuximab-based regimens have shown efficacy in 1q21^+^ RRMM patients. In the ICARIA-MM trial, Isa-Pd significantly improved PFS (9.5 months vs. 3.8 months; HR = 0.40, 95% CI: 0.25-0.63) and OS (21.3 months vs. 13.9 months) compared to Pd in patients with 1q21^+^.^97^ The IKEMA trial further confirmed the benefit, with Isa-Kd extending PFS to 25.8 months vs. 16.2 months (HR, 0.58, 95% CI: 0.37-0.92).^98^ Higher MRD negativity rates were also observed with Isa-Kd in different 1q21^+^ copies with or without high-risk chromosomal abnormality, reinforcing its potential to overcome the poor prognosis associated with 1q21^+^. Furthermore, the subgroup analysis indicated that Isa-Kd was also effective in patients with ≥4 copies of 1q21^+^, as demonstrated in improved PFS compared to Kd alone (HR, 0.73, 95% CI: 0.33-1.63), suggesting potential to mitigate the adverse prognosis associated with 1q21 amplification.

Isatuximab was well tolerated across risk groups. Although grade ≥3 AEs were more frequent with Isa-Pd in both high-risk (95.7% vs. 67.6%) and standard-risk groups (85.4% vs. 76.3%), SAE rates remained acceptable.^99^ Importantly, isatuximab did not increase treatment discontinuations, with lower discontinuation rates in the high-risk subgroup (8.7% vs. 23.5%). Common TEAEs in high-risk patients include febrile neutropenia (13% vs. 0%) and pneumonia (21.7% vs. 17.6%), with manageable G-CSF use (28.1% vs. 36.8%). These findings support the use of isatuximab in 1q21^+^ RRMM with sustained efficacy and manageable safety

#### Conclusion

Anti-CD38-based therapies are appropriate for RRMM patients with 1q21^+^ cytogenetic abnormalities.

#### Level of Consensus

100% (17) agree.

Total: 17 voters.

### Chapter 3: Safety profiles and management in anti-CD38 regimens

The integration of anti-CD38 mAbs into RRMM regimens has significantly improved clinical outcomes, but also raised particular safety concerns that may require specific monitoring and proactive management. Across phase III trials, common AEs include hematologic toxicities such as neutropenia and thrombocytopenia, IRRs, infections, and potential cardiovascular complications in specific combinations.^32–35^

In the ICARIA-MM trial, Isa-Pd was associated with higher grade ≥ 3 AEs compared to Pd (86.8% vs. 70.5%), notably neutropenia (50% vs. 35%), pneumonia (23% vs. 21%), and thrombocytopenia (13% vs. 12%).^35,99^ In IKEMA trial, Isa-Kd presented a higher incidence of grade ≥3 AEs (83.6% vs. 73.0%), with increased rates of anemia (24.3% vs. 21.3%), neutropenia (20.4% vs. 7.4%), thrombocytopenia (30.0% vs. 23.8%), and pneumonia (18.6% vs. 12.3%).^33,47^ The CANDOR trial demonstrated increased grade ≥3 AEs with Dara-Kd versus Kd (88.6% vs. 78.4%), including thrombocytopenia (24.7% vs. 16.3%), neutropenia (10.1% vs. 6.5%), infections (46% vs. 32%), and hypertension (23.4% vs. 17.6%).^32^ Likewise, in APOLLO, neutropenia (69% vs. 51%) and infections (28% vs. 23%) were more frequent with Dara-Pd than Pd.^34,48^

Meta-analyses confirmed that anti-CD38 therapies significantly increase the risk of neutropenia and thrombocytopenia.^100–102^ For thrombocytopenia, the relative risk (RR) was 1.301 (95% CI: 1.055-1.603, 95% prediction interval [PI]: 0.663-2.554; I² = 86.4%, Tau² = 0.0575); for neutropenia, the RR was 1.441 (95% CI: 1.315-1.579, 95% PI: 1.154-1.740; I² = 18.5%, Tau² = 0.0036). Subgroup analyses further supported elevated hematologic toxicity risks with daratumumab- and isatuximab-based combinations, particularly those involving IMiDs or PIs.

Despite increased grade ≥3 AEs, discontinuation rates due to toxicity were lower (Isa-Pd and Pd [7.2% vs. 12.8%], Isa-Kd and Kd [8.5% vs. 13.9%], Dara-Kd and Kd [22% vs. 25%], and Dara-Pd and Pd [2% vs. 3%]), indicating overall manageable and safety profiles.^103^ These results reinforced the importance of AE monitoring and supportive care in patients receiving anti-CD38-based therapies. A summary of the incidence of specific AEs and managements in patients with RRMM who received anti-CD38-based therapies from the phase III trials is provided in **Table 3**.

**Table 3.** Common AEs and Management Approaches

*Adverse Events*	*Incidence of ≥3 AEs*	*Approach*
Neutropenia	10.1% - 69%	Regular complete blood count (CBC) monitoring Recombinant granulocyte colony-stimulating factor (G-CSF) support should be considered for grade ≥3 neutropenia Dose interruption or modification
Thrombocytopenia	13% - 30%	Regular CBC monitoring Withhold or adjust treatment doses Consider transfusion for severe thrombocytopenia Discontinue or modify concurrent medications that may exacerbate thrombocytopenia Ensure adequate intake of nutrients essential for platelet production
Anemia	17.5% - 25%	Regularly assess hemoglobin levels and anemia symptoms Consider erythropoiesis-stimulating agents (ESAs), especially in in symptomatic anemia Consider red blood cell (RBC) transfusion for immediate symptom relief in severe anemia
Infections	24.3% - 46%	Prophylactic antibiotics to reduce the incidence of grade ≥3 infections Consider antiviral and antifungal prophylaxis for high-risk patients Administer intravenous immunoglobulin (IVIG) for patients with recurrent infections and hypogammaglobulinemia Use G-CSF for neutropenia prevention in patients receiving myelosuppressive therapy Ensure influenza, pneumococcal, and COVID-19 vaccines to prevent viral and bacterial infections

Is there agreement that hematologic toxicities are among the most common adverse events in patients receiving anti-CD38-based therapy, and that their management typically involves routine complete blood count (CBC) monitoring, dose adjustments, or granulocyte colony-stimulating factor (G-CSF) support?

#### Statement

Hematologic toxicities are among the most common adverse events in patients receiving anti-CD38-based therapy, and that their management typically involves routine CBC monitoring or G-CSF support (A1).

#### Discussion

##### Hematologic Abnormalities

Hematologic toxicities, particularly neutropenia, thrombocytopenia, and anemia, are common in RRMM patients receiving anti-CD38-based therapies in combination with PIs/IMiDs. These can significantly impact treatment adherence and patient quality of life, necessitating proactive management strategies:

1. Neutropenia: Regular CBC monitoring is crucial for early detection, as neutropenia-related infections can lead to treatment delays or dose modifications that compromise efficacy.^104^ G-CSF is the standard management approach and should be considered for grade ≥ 3 neutropenia to prevent febrile neutropenia.^105^ Persistent or chronic neutropenia despite G-CSF administration may require prophylactic antibacterial, antifungal, or antiviral therapy. In accordance with FDA label recommendations for PIs, IMiDs, and anti-CD38 mAbs, treatment should be withheld or delayed if absolute neutrophil count falls below 0.5 × 10⁹/L or febrile neutropenia is present, and resumed when neutrophil recovers to 1 × 10⁹/L. IMiDs dose reductions are recommended for recurrent events.
2. Thrombocytopenia: Caused by cytotoxic effects on megakaryocytes, thrombocytopenia increases bleeding risk. At least weekly CBC monitoring enables early detection and intervention. According to FDA labels for PIs and IMiDs, treatment should be withheld or adjusted if platelet counts fall below 10 × 10⁹/L or bleeding occurs.
3. Anemia: Found in over 40% of RRMM patients, anemia results from marrow suppression, MM-related effects, or renal impairment.^106^ Treatments for anemia include red blood cell (RBC) transfusion for short-term support and erythropoiesis-stimulating agents (ESAs, e.g., epoetin alfa, darbepoetin alfa) for sustained hemoglobin improvement.^107^ However, ESAs is associated with increased thromboembolic risk, particularly when used with PIs, IMiDs and dexamethasone.^108–112^ Therefore, careful monitoring is essential. ESAs should be considered in patients with symptomatic anemia.

In conclusion, hematologic toxicities such as neutropenia, thrombocytopenia, and anemia are common in RRMM patients receiving anti-CD38-based combinations with PIs or IMiDs. To optimize treatment adherence and efficacy, dose modifications should be applied to IMiDs, and the dosing intervals for PIs, IMiDs, or anti-CD38 mAbs may be adjusted as needed to allow for sufficient hematologic recovery. With proactive supportive care such as G-CSF and antimicrobial prophylaxis, cytopenia does not necessarily increase infection risk. Careful monitoring and individualized management remain essential to minimize treatment-related complications and improve patient outcomes.

#### Conclusion

Hematologic toxicities are among the most common adverse events in patients receiving anti-CD38-based therapy, and that their management typically involves routine CBC monitoring or G-CSF support.

#### Level of Consensus

94% (16) agree; 6% (1) neutral.

Total: 17 voters.

Is it generally recommended that prophylactic antiviral therapy, antibacterial agents, and vaccination against infections be administered before initiating anti-CD38-based therapy?

#### Statement

Patients treated with anti-CD38-based therapy are at increased risk of severe infections. Consequently, prophylactic antiviral therapy, antibacterial agents, and vaccination against infections are advised prior to initiating anti-CD38-based therapy (A1).

#### Discussion

##### Infections

Anti-CD38-based therapies have significantly improved RRMM outcomes, but are associated with increased infection risks because of immunosuppressive effects.^113^ Consequently, guidelines from the IMWG and NCCN Guidelines on Prevention and Treatment of Cancer-Related Infections specifically emphasize infection prevention strategies, including baseline screening for latent viruses (e.g., cytomegalovirus, hepatitis B virus, Epstein-Barr virus) and routine CBC monitoring for neutropenia or lymphopenia.^114^ Additionally, patients should undergo pulmonary function assessments and imaging (e.g., chest X-ray or computed tomography scan) in cases of suspected pneumonia or respiratory complications. Empirical broad-spectrum antibiotics should be initiated promptly in patients presenting with febrile neutropenia, and early microbiological workups should guide targeted antimicrobial therapy, especially in heavily pretreated patients with prolonged lymphopenia.

Prophylactic antimicrobial strategies are strongly recommended. Acyclovir or valacyclovir is standard for all patients to prevent herpes zoster and simplex virus reactivation. Trimethoprim-sulfamethoxazole prophylaxis is recommended for *Pneumocystis jirovecii* pneumonia (PJP) prevention in patients receiving high-dose dexamethasone or with severe, prolonged neutropenia. In high-risk patients with severe neutropenia, levofloxacin prophylaxis may be used. Inactivated influenza, pneumococcal (PCV13 and PPSV23), and recombinant zoster vaccines are recommended before starting therapy. Antifungal prophylaxis with fluconazole is advised, escalating to agents like posaconazole during prolonged neutropenia.

Anti-CD38 mAbs may induce secondary hypogammaglobulinemia, because of plasma cell depletion.^115^ Regular serum IgG monitoring is recommended, with prophylactic antibiotics considered when IgG levels fall below 400 mg/dL. For IgG less than 200 mg/dL, or with recurrent or severe infections, immunoglobulin replacement therapy, either intravenous or subcutaneous, is recommended. Particularly, when IgG drops below 100-200 mg/dL, prompt initiation of immunoglobulin replacement therapy should be strongly considered regardless of infection history. These preventive strategies, combined with patient education on infection risk and prompt symptom recognition, are essential to minimize infection-related complications and support anti-CD38-based treatment adherence in RRMM. The multifaceted approach to mitigate the risk of infections is summarized in **Table 4**.

**Table 4.** Approaches for prophylaxis of infections

*Infection Type*	*Common Pathogens*	*Monitoring Recommendations*	*Approaches*
Bacterial	*Streptococcus pneumoniae*, *Escherichia coli, Pseudomonas aeruginosa*	CBC with differential blood cultures in febrile patients respiratory assessment chest imaging if pneumonia suspected	Levofloxacin for high-risk neutropenic patients IVIG for recurrent bacterial infections and hypogammaglobulinemia Vaccination with PCV13 and PPSV23 recommended Consider antibiotic prophylaxis during severe neutropenia
Viral	Cytomegalovirus (CMV), Herpes simplex virus (HSV), Varicella-zoster virus (VZV), Hepatitis B virus (HBV)	Serologic screening for CMV, HBV, and EBV before therapy; PCR-based monitoring in high-risk cases	Acyclovir or valacyclovir for herpes simplex/zoster, HBV antiviral therapy for patients with active or prior HBV infection Patients should receive influenza and recombinant zoster vaccines before therapy initiation
Fungal	Candida species, Aspergillus species, Cryptococcus neoformans	Fungal biomarkers (galactomannan, beta-D-glucan) Periodic chest imaging Fungal culture if infection suspected	Fluconazole for candidiasis Posaconazole or voriconazole for prolonged neutropenia or risk of invasive fungal infection High-risk patients (e.g., prolonged neutropenia) may require prophylactic antifungal therapy
Opportunistic	*Pneumocystis jirovecii*, Mycobacterium tuberculosis, *Toxoplasma gondii*	CD4 count assessment in severely immunosuppressed patients Screening for latent tuberculosis in high-risk populations	Trimethoprim-sulfamethoxazole (TMP-SMX) for PJP Alternative prophylaxis (dapsone, atovaquone) for allergic patients Monitor for reactivation of latent infections, especially in patients receiving high-dose corticosteroids or prolonged immunosuppression

#### Conclusion

Patients treated with anti-CD38-based therapy are at increased risk of severe infections. Consequently, prophylactic antiviral therapy, antibacterial agents, and vaccination against infections are advised prior to initiating anti-CD38-based therapy.

#### Level of Consensus

82% (14) agree; 18% (3) neutral.

Total: 17 voters.

### Chapter 4: Integration of novel targets with anti-CD38-based therapies

Are anti-CD38 mAbs in combination with different targeted therapies recommended for multiple-drug regimens for the treatment of RRMM?

#### Statement

Anti-CD38 mAbs in combination with different targeted therapies are recommended as part of multi-drug regimens for the treatment of RRMM (B1).

#### Discussion

The combination of anti-CD38 mAbs with novel agents beyond traditional PIs and IMiDs is expanding treatment options for RRMM. These regimens aim to enhance anti-myeloma efficacy by leveraging distinct mechanisms of action, such as selective nuclear export inhibition with selinexor, and T-cell redirection with BsAbs like talquetamab, teclistamab, and linvoseltamab.^116–119^ Additionally, the combination of elotuzumab with isatuximab (Isa-EloPD) targets both SLAMF7 (signaling lymphocytic activation molecule family member 7) and CD38, offering a unique immune-mediated therapeutic approach.^120^ These emerging regimens reflect a shift toward multimodal myeloma therapy, potentially improving responses in patients who have exhausted standard PI- and IMiD-based treatments.

##### Selinexor, Daratumumab, Bortezomib and Dexamethasone (S-DVd)

Selinexor is a first-in-class oral exportin-1 (XPO1) inhibitor that retains tumor suppressor proteins within the nucleus, leading to cell cycle arrest and apoptosis in malignant plasma cells.^121,122^ It has been approved for RRMM in combination with bortezomib and dexamethasone (SVd) based on the BOSTON trial.^123^ To further enhance efficacy, the GEM-SELIBORDARA phase II study investigated the combination of selinexor with daratumumab, bortezomib, and dexamethasone (S-DVd) in 57 RRMM patients.^117^ In the heavily pretreated cohort (≥ 3 prior lines), the ORR was 50% with a median PFS of 7 months; in early-relapse patients (≥ 1 prior line), ORR reached 82%, with a median PFS of 24 months. Lenalidomide-refractory patients presented a median PFS of 22.1 months, suggesting benefits in IMiD-resistant disease. However, toxicity remained a challenge, with thrombocytopenia (69%) and nausea (38%) being the most frequent AEs, leading to dose modifications in 62% of patients. Despite not meeting its primary endpoint, S-DVd demonstrated promising clinical activity and may serve as an alternative regimen for RRMM patients, particularly those requiring a quadruplet therapy approach.

##### Isatuximab, Elotuzumab, Pomalidomide and Dexamethasone (Isa-EloPD)

Elotuzumab is a humanized IgG1 mAb targeting SLAMF7, a surface marker co-expressed with CD38 on most MM cells, enhancing NK cell-mediated cytotoxicity.^124,125^ The phase II IMPEDE study (NCT04835129) evaluated the combination of isatuximab, elotuzumab, pomalidomide, and dexamethasone (Isa-EloPD) in RRMM patients previously treated with lenalidomide and a PI.^126^ Among 15 patients enrolled, the ORR was 80%, with six patients achieving a VGPR or better, and a 12-month PFS rate of 67%. The combination was well tolerated, with no dose-limiting toxicities. Common grade 3-4 AEs included lymphopenia (93%), neutropenia (93%), and leukopenia (40%), while grade 3-4 infections occurred in only one patient. Importantly, NK cell counts declined, but cytotoxic function was preserved, supporting the feasibility of dual SLAMF7 and CD38 blockade in RRMM. These findings suggest Isa-EloPD is a safe and effective regimen, warranting further investigation in larger studies.

##### Talquetamab, Daratumumab ± Pomalidomide

Talquetamab is a first-in-class BsAb targeting G protein-coupled receptor class C group 5 member D (GPRC5D) and CD3, redirecting T-cells to eliminate MM cells independently of BCMA expression.^127,128^ It has been approved for RRMM patients with ≥4 prior therapies based on MonumenTAL-1.^129^ The TRIMM-2 trial evaluated subcutaneous talquetamab plus daratumumab (Tal-Dara) in RRMM patients with ≥3 prior therapies, including PIs and IMiDs.^130^ Of the 65 patients, 78% achieved an ORR, with 66% achieving VGPR or better and 45% reaching CR. Responses were durable, with 86% of responders maintaining responses at 12 months. The median PFS was 19.4 months; 12-month PFS and OS rates were 76% and 93%, respectively. Tal-Dara was generally well tolerated, with common AEs including cytokine release syndrome (78%, all grade 1-2), dysgeusia (75%), neutropenia (38%), and infections (63%), including 22% grade 3-4 and two grade 5 pneumonia-related deaths. These results suggest that Tal-Dara as a potential option for heavily pretreated RRMM, including those refractory to anti-CD38 and BCMA therapies. The ongoing MonumenTAL-3 trial is evaluating the efficacy and safety of Tal-Dara with or without pomalidomide (Tal-DP or Tal-D) versus Dara-Pd in patients with RRMM who have received at least one prior line of therapy.^118^

##### Teclistamab, Daratumumab, and pomalidomide

Teclistamab, the first approved BsAb targeting BCMA and CD3, has demonstrated strong efficacy in triple-class exposed RRMM.^131^ In MajesTEC-2 and TRIMM-2 trials, teclistamab was combined with daratumumab and pomalidomide (tec-DP) in 27 RRMM patients.^132^ With a median follow-up of 25.8 months, the ORR was 88.5%, with 84.6% achieving VGPR or better, and 61.5% CR or better. The median time to first response was 0.95 months, and the median PFS was 26.5 months. Safety findings included neutropenia (77.8%), cough (59.3%), cytokine release syndrome (55.6%, mostly grade 1-2), and infections (92.6%), with 63% experiencing grade 3-4 infections. Importantly, all fatal infections occurred before intensified prophylaxis strategy, including intravenous immunoglobulin supplementation, after which, no further fatal infections occurred. These results support tec-DP as a feasible and potent regimen for heavily pretreated RRMM, with appropriate prophylactic measures improving tolerability.

##### Linvoseltamab with Daratumumab

Linvoseltamab is a BsAb that targets BCMA and CD3, engaging T-cells to induce targeted cytotoxicity against MM cells.^133,134^ The phase I/II LINKER-MM1 trial evaluated linvoseltamab monotherapy in RRMM patients who had progressed after at least three prior lines of therapy, including a PI, an IMiDs, and an anti-CD38 mAb. In patients receiving the recommended phase II dose of 200 mg, the ORR was 71%, with 50% achieving a CR or better, and the median duration of response was 29.4 months. The PFS was NR, and the 12-month OS was 75.3%.^135^ Given its promising response rates in triple-class refractory RRMM patients, linvoseltamab has been explored in combination with daratumumab to enhance immune-mediated tumor clearance.^119^

Although combinations of anti-CD38 mAbs with novel and targeted therapies have shown promising efficacy and manageable safety profiles, larger clinical trials are needed to further validate their effectiveness in RRMM patients.

#### Conclusion

Anti-CD38 mAbs in combination with different targeted therapies are recommended as part of multi-drug regimens for the treatment of RRMM.

#### Level of Consensus

100% (17) agree.

Total: 17 voters.

## Discussion

The emergence of anti-CD38 mAbs, such as daratumumab and isatuximab, has significantly reshaped the therapeutic landscape of RRMM. Building upon the successful integration of anti-CD38 mAbs into frontline regimens for NDMM, the RRMM setting has also benefited from their use in diverse triplet and emerging quadruplet regimens.^57^ In this consensus, we evaluated the role of anti-CD38-based therapies in RRMM, especially considering clinical efficacy across subgroups, rechallenge strategies, special populations (e.g., renal impairment, elderly), and safety management.

In patients with RRMM, particularly those who are refractory to lenalidomide and/or bortezomib, anti-CD38-based triplet regimens have demonstrated substantial improvements in PFS, ORR, and MRD negativity rates compared to backbone doublets. Landmark phase III trials, including CANDOR (Dara-Kd), IKEMA (Isa-Kd), APOLLO (Dara-Pd), and ICARIA-MM (Isa-Pd), have established the clinical benefit of these regimens across multiple prior lines of therapy and high-risk cytogenetic profiles such as 1q21^+^.^32–35^ Notably, Isa-Kd and Dara-Kd significantly improved median PFS and OS, compared to Kd alone. Dara-Pd and Isa-Pd also significantly improved outcomes in lenalidomide-refractory populations, who have limited treatment options. Additionally, MRD negativity has become an important treatment goal. Anti-CD38-based therapies have demonstrated higher MRD− rates compared to control arms, and future practice may incorporate MRD status to guide treatment duration, switch decisions, and maintenance strategies in RRMM.

The consensus also explored the feasibility and rationale of anti-CD38 mAb rechallenge. While cross-resistance remains a concern, emerging evidence suggests that responsiveness may regain following a sufficient treatment-free interval, possibly because of partial recovery of CD38 expression and immune effector function.^60^ A washout interval of at least 6 to 12 months and higher CD38 receptor density on myeloma cells have been associated with better outcomes upon rechallenge.^61,62^ Isatuximab after prior daratumumab (Dara-Isa), from phase II and real-world studies, has demonstrated clinical benefit in some patients, particularly with sufficient washout periods and favorable biomarker profiles. Despite the incorporation of anti-CD38 mAbs into NCCN and ESMO guidelines, several gaps remain—including optimal sequencing, re-treatment feasibility, and the specific role of different anti-CD38 mAbs in resistant disease. Daratumumab rechallenge (Dara-Dara) may also be effective after prior durable response and a washout period. However, switching from isatuximab to daratumumab (Isa-Dara) is less studied. Anti-CD38 mAb rechallenge should be approached cautiously and is best considered as a bridge strategy to definitive therapies such as CAR-T or BsAbs in patients with limited options. Prospective studies are required to better define optimal conditions and biomarkers for guiding rechallenge decisions.

Special populations such as those with renal impairment or advanced age require careful therapeutic considerations. Anti-CD38-based regimens, particularly Dara/Isa combined with pomalidomide or carfilzomib, have shown favorable efficacy and manageable safety in patients with moderate renal impairment (eGFR 30-60 mL/min/1.73 m²), without requiring dose adjustments of the anti-CD38 mAb.^136^ Elderly and frail patients also benefit from the tolerability of anti-CD38 regimens. Subgroup analyses from ICARIA-MM demonstrated solid clinical benefit with acceptable safety of Isa-Pd in patients aged over 75 years.^82^ For daratumumab, the subgroup analyses specifically focusing on patients aged ≥75 years are demonstrated in POLLUX and CASTOR trials.^80^ In the APOLLO trial, Dara-Pd demonstrated consistent PFS benefit and low discontinuation rates in older patients (≥ 75 years), despite the absence of formal frailty scoring. The inclusion of patients with reduced performance status and favorable tolerability further supports its use in this group. Similarly, while CANDOR lacked detailed data for patients ≥ 75 years, the overall safety profile and subcutaneous formulation of daratumumab enhance its practicality in elderly or frail populations.^137,138^

In terms of safety, hematologic toxicities, including neutropenia and anemia, remain the most common AEs.^139^ Management strategies include routine complete blood count monitoring, supportive use of G-CSF, and dose modifications of IMiDs or PIs rather than discontinuation of the anti-CD38 mAb. Infections, especially viral reactivation and pneumonia, are also common; thus, prophylactic antivirals (e.g., acyclovir), pneumococcal and influenza vaccinations, and early infection management are recommended. Infusion-related reactions (IRRs) are mostly low grade and decrease after the first dose, particularly with subcutaneous (SC) formulations like SC daratumumab. Ongoing trials investigating SC isatuximab in combination with Pd or Kd have reported interim results showing no IRR with SC Isa-Pd, while maintaining safety and efficacy comparable to that observed in the Phase III ICARIA-MM trial.^140,141^ Further evaluation of the efficacy and safety of SC isatuximab is underway in the randomized, multinational, noninferiority Phase III IRAKLIA/EFC15951 trial (NCT05405166), which compares SC administration via on-body delivery system (OBDS) to IV infusion. The study investigates SC isatuximab in combination with Pd in patients with RRMM who have received at least one prior line of therapy.

The integration of anti-CD38 therapy with newer agents such as selinexor and BsAbs (e.g., elotuzumab, teclistamab, and talquetamab) offers promising future directions. In addition, preliminary data from early-phase trials (e.g., Dara-KPd, Isa-KPd, Isa-EloPd) demonstrated promising efficacy, especially in lenalidomide- and PI-refractory populations. The consensus supports the further development of such combinations, aligned with NCCN endorsed regimens such as KPd and EloPd triplets. In later lines of therapy, BCMA-targeted CAR-T therapies (e.g., ciltacabtagene autoleucel (cilta-cel), idecabtagene vicleucel (ide-cel)) and BsAbs (e.g., teclistamab, elranatamab, talquetamab) have demonstrated high efficacy in triple-class-exposed RRMM. The CARTITUDE-4 and KarMMa-3 trials demonstrated superior PFS with CAR-T compared to standard regimens, including those containing anti-CD38 mAbs (e.g., Dara-Pd).^142,143^ Meanwhile, BsAbs have emerged as a promising intermediate option. Teclistamab achieved a median PFS of 11.3 months in adults with triple-class-exposed RRMM, who have received ≥ 3 prior lines of therapy; talquetamab reported ORRs of 64-70%, and elranatamab demonstrated a 61% ORR with a 15-month PFS rate of 50.9%.^129,131,144^ While CAR-T and BsAbs offer deeper responses, they are associated with cytokine release syndrome, neurotoxicity, and infection risks, requiring specialized care. Anti-CD38-based therapies remain an important option, offering favorable efficacy and manageable safety, especially for patients in earlier relapse or those ineligible for cellular therapies.

Treatment selection in RRMM should be individualized based on prior treatment exposure, patient frailty, disease aggressiveness, and healthcare resources. In early relapses without triple-class refractoriness, anti-CD38-based regimens remain a preferred standard for balancing efficacy and safety. For triple-class-refractory patients, CAR-T therapies (clita-cel, ide-cel), offer the deepest and most durable responses and are preferred for fit patients with access to specialized centers. BsAbs serve as effective “off-the-shelf” options for patients ineligible for or awaiting CAR-T therapy but require careful infection monitoring. Taken together, tailoring therapy to clinical and logistical factors is essential for durable disease control and improved survival.

Overall, the expert panel supports the use of anti-CD38-based regimens as a well-established option in RRMM across diverse clinical scenarios. Their integration into personalized treatment strategies, rechallenge recommendations, and combination regimens with emerging agents holds promise for further improving patient outcomes. While all 17 panelists involved in this consensus are based in the Asia-Pacific region, the recommendations reflect regional clinical practices, treatment access, and healthcare infrastructure. We acknowledge that treatment paradigms, drug availability, and reimbursement policies may differ across global healthcare systems. Accordingly, these recommendations should be interpreted in the context of local practice. Further international collaboration and validation will be important to refine and adapt these insights for broader global applicability.

### Authors’ Contribution

Conceptualization: Wenming Chen; Resources: Wenming Chen, Zhen Cai, Chor Sang Chim, Wee Joo Chng, Juan Du, Chengcheng Fu, Wen Gao, Ichiro Hanamura, Jian Hou, Jeffrey Shang-Yi Huang, Tadao Ishida, Chunrui Li, Aijun Liu, Vadim Ptushkin, Naoki Takezako, Raymond Siu Ming Wong, Dok Hyun Yoon; Writing – original draft: Wenming Chen; Writing – review & editing: Zhen Cai, Chor Sang Chim, Wee Joo Chng, Juan Du, Chengcheng Fu, Wen Gao, Ichiro Hanamura, Jian Hou, Jeffrey Shang-Yi Huang, Tadao Ishida, Chunrui Li, Aijun Liu, Vadim Ptushkin, Naoki Takezako, Raymond Siu Ming Wong, Dok Hyun Yoon.

### Competing of Interest – COPE

WJC has received payment or honoraria for lectures, presentations, speakers bureaus, manuscript writing, or educational events from J&J, Sanofi, BMS, GSK, Takeda, and Regeneron, and stock or stock options from Kyan Technologies and Medicia.ai. IH has received payment or honoraria for lectures, presentations, speakers bureaus, manuscript writing, or educational events from Takeda, BMS, Ono, Janssen, Sanofi, and Pfizer. TI has received payment or honoraria for lectures, presentations, speakers bureaus, manuscript writing, or educational events from Takeda, BMS, Ono, CSL Behring, Janssen, Sanofi, and Pfizer. RSMW has received medical writing support from Sanofi, and payment or honoraria for lectures, presentations, speakers bureaus, manuscript writing, or educational events from Bistol-Myer-Squibb, GSK, Sanofi, Pfizer, Novartis, and AstraZeneca, and support for attending meetings and/or travel from Beigene, Sanofi, and Novartis, and participation on a Data Safety Monitoring Board or Advisory Board from Novartis, J&J, GSK, Sanofi, and Roche. DHY has received grants or contracts from any entity from Abbvie, BeOne, Boryung, Celltrion, Kyowa Kirin, Janssen, Samyang, and Sanofi, and consulting fees from Abclon, BeOne, BMS, GI Cell, GC Cell, Verismo, J&J, Novartis, and Roche, and payment or honoraria for lectures, presentations, speakers bureaus, manuscript writing, or educational events from BMS, Boryung, GSK, Kyowa Kirin, Novartis, Roche, Takeda, and J&J, and patents planned, issued, or pending from Boryung. WC, ZC, CSC, JD, CF, WG, JH, JSYH, CL, AL, VP, and NT have declared no conflicts of interest.

### Ethical Conduct Approval – Helsinki – IACUC

Not applicable.

### Informed Consent Statement

All authors and institutions have confirmed this manuscript for publication.

## Data Availability

Not applicable.
